# Efficacy and Complications of Life-Control Portable Resuscitator for Transport of In-Hospital Patients

**DOI:** 10.1155/2019/8282039

**Published:** 2019-07-25

**Authors:** Mustafa Ahmet Afacan, Mehmet Özgür Erdogan, Abdullah Algın, Miraç Kırcı, Sahin Colak

**Affiliations:** ^1^Department of Emergency Medicine, University of Health Sciences, Haydarpasa Numune Training and Research Hospital, Istanbul, Turkey; ^2^Department of Emergency Medicine, Bahcesehir University, Istanbul, Turkey; ^3^Department of Emergency Medicine, University of Health Sciences, Umraniye Training and Research Hospital, Istanbul, Turkey

## Abstract

**Aim:**

Equipment used for in-hospital patient transfers should be safe for the patient, inexpensive, and easy to use. Disposable mechanical ventilators are a reasonable choice for in-hospital transfers. Life-control Portable Resuscitator (LPR) is a gas-powered automatic resuscitator designed for short-term ventilation during the transport of critically ill mechanically ventilated patients. The aim of this study was to investigate the efficacy and safety of, and complications associated with, the LPR.

**Materials and Methods:**

A total of 77 (age > 18 years) critically ill mechanically ventilated emergency service patients transported to in-hospital units with an LPR were included in this study. Categorical variables are presented as frequencies (numbers and percentages), and continuous variables are presented as means ± standard deviation with corresponding 95% confidence intervals (CIs). Paired-sample t-tests were used to analyze normally distributed variables.

**Results:**

Vital signs showed no significant difference after transport. After transport mean pH, PaCO2, and lactate levels of all patients increased statistically significantly and approached normal range values. PaO2 levels increased significantly after transport. HCO3, PIP, and BE showed no significant difference after transport. Device-related complications during transport included O_2_ cable disconnection (11.6%), device failure (2.59%), vomiting (1.2%), and extubation (2.59%).

**Conclusion:**

In our study group, the LPR was reliable according to the vital signs and blood gas analyses, although these devices should be used only by skilled personnel due to the possible risk of complications during transport.

## 1. Introduction

Interhospital patient transfers are a routine and essential part of the care of many patients. Moreover, in-hospital transport of patients to tomography, magnetic resonance imaging (MRI), and angiography units is a routine and essential part of care for critically ill, emergency service, and intensive care unit (ICU) patients. However, transport of a patient within a hospital and between medical facilities without interrupting medical treatment can be a challenging task. Indeed, ICU ventilators are not designed for transport and their size, weight, and need for a power supply limit their use in the context of patient transport [1]. These factors also restrict their use in emergency departments.

The transportation of critically ill patients requiring mechanical ventilation is recognized as a high-risk and expensive procedure. Equipment used for in-hospital patient transfers, which should be safe for the patient, inexpensive, and easy to use, include manual bag-type valve resuscitators and expensive portable transport ventilators. Moreover, disposable mechanical ventilators are a reasonable choice for in-hospital transfers. There are sophisticated transport ventilators such as Uni-Vent Eagle 754, VersaMed iVent, Newport HT50, and Pulmonetic Systems LTV 1000. There are simple transport ventilators such as Oceanic Medical Products Magellan, Bio-Med Devices IC2A, Pneupac ParaPAC Medic, Pneupac ParaPAC Transport 200D, Life Support Products Auto Vent 2000, CAREvent ATV+, Vortran RespirTech Pro, Percussionaire TXP, Bio-Med Devices Crossvent 3, Bird Avian, and Pneupac ComPAC 200. These devices operate with electric power supply, compressed gas, or both. All these devices have various advantages and disadvantages. Improvement of these single use automatic ventilators may help in cheaper and safer patient transport inside and outside the hospital. The Life-control Portable Resuscitator (Egemen International-TMT Medical Products, Gaziemir, Izmir, Turkey) is a gas-powered automatic resuscitator designed for short-term ventilation of critically ill mechanically ventilated patients during transport. The aim of this study was to investigate the efficacy and safety of, and complications associated with, the LPR.

## 2. Materials and Methods

Critically ill mechanically ventilated emergency service patients admitted to the Department of Emergency Medicine of Haydarpasa Numune Training and Research Hospital between January and December 2017 were included in this study.

A total of 77 (age > 18 years) critically ill mechanically ventilated emergency service patients transported to in-hospital units with an LPR were included in this study. Of these, 33 were type 1 respiratory failure patients and 44 were type 2 respiratory failure patients with a transport duration exceeding 30 minutes. Each patient received 2 mg/kg IV bolus ketamine and 50 mcg/kg/min infusion for sedation and mechanical ventilation. Ketamine was used because its side effect profile was lower and provides better hemodynamic stability than other anesthetic agents. Age, gender, vital signs, date and time of admission, type of respiratory failure, arterial blood gas parameters, and complications were recorded on standardized forms. This prospective study was approved by the institutional ethics committee of Haydarpasa Numune Training and Research Hospital (HNEAH-KAEK2016/KK/85). Informed consent was obtained from the relatives of all participants.

Hypoxemic respiratory failure (type I) is defined as an arterial oxygen tension (PaO_2_) < 60 mmHg with a normal or low arterial carbon dioxide tension (PaCO_2_). Hypercapnic respiratory failure (type II) is characterized by a PaCO_2_ > 50 mmHg and is associated with hypoxia.

Exclusion criteria included a history of surgery or trauma, PaO2/ fraction of inspired oxygen (FiO2) < 100, mean arterial pressure (MAP) < 60 mmHg, cardiopulmonary resuscitation, a central line inserted in the subclavian or internal jugular vein, body mass index > 35 kg/m^2^, and age < 18 years.

### 2.1. Device Definition

**Figure 1:**
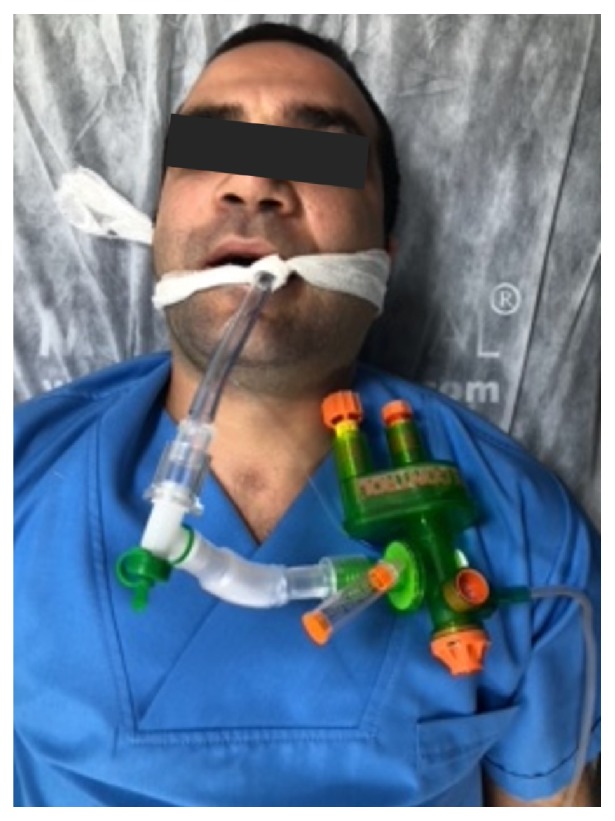
Demonstration of the device.

The working principle of LPR is pressure-cycled ventilation, and the device provides ventilatory support in pressure control mode. The LPR runs on a continuous flow of gas of up to 40 L/min. Exhalation starts after peak pressure is reached during the inhalation period, and inhalation starts after the exhalation pressure drops to the positive end-expiratory pressure (PEEP). If the LPR is connected to a 75 pound per square inch gauge (PSIG) high-flow source, the device delivers a flow of 40 L/min. Peak inspiratory pressure (PIP) can be adjusted to 15–45 cmH_2_0. PEEP approaches a standard 20% of PIP level. PEEP is automatically adjusted by the device and cannot be changed externally by the user. Device is shown in Figure 1 (CE-2292).

### 2.2. Statistical Analysis

Categorical variables are presented as frequencies (numbers and percentages), and continuous variables are presented as means ± standard deviation (SD) with corresponding 95% confidence intervals (CIs). Paired-sample t-tests were used to analyze normally distributed variables. All statistical analyses were performed using SPSS for Windows (ver. 20.0; SPSS Inc., Chicago, IL, USA). A p-value < 0.05 was considered to indicate statistical significance.

## 3. Results

Data from 45 male and 32 female patients were analyzed. The mean age of the patients was 73.7 ± 14.51 years (CI 70.5–77.04 years).

The initial MAP of all patients was 69.16 ± 4.78 mm Hg while that after transport was 69.09 ± 4.18 mm Hg (p = 0.898), and the mean initial heart rate (HR) was 99.82 ± 11.64 beats/min while that after transport was 99.34 ± 12.33 beats/min (p = 0.412), which were not significantly different. The respiratory rate (RR) did not significantly change between baseline and posttransport (14.44 ± 2.54 and 14.6 ± 2.11 breaths/min, respectively; p = 0.453). All vital sign measurements are presented in Table 1.

The blood pH of all patients was 7.259 ± 0.197 at baseline and increased significantly to 7.315 ± 0.169 after transport (p = 0.001). Moreover, the initial PaO_2_ level and that after transport were 100.837 ± 75.824 and 274.828 ± 150.647, respectively, which was significantly different (p< 0.05). The mean PaCO_2_ level significantly decreased from 51.732 ± 25.114 at baseline to 41.866 ± 16.262 after transport (p< 0.05).

The mean initial HCO_3_ level was 51.732 ± 25.114 and that after transport was 41.866 ± 16.262 (no significant difference; p = 0.102). The mean initial lactate level (5.340 ± 4.705) was significantly higher than that after transport (4.566 ± 4.331) (p = 0.047). The mean BE level at baseline and after transport was -4.554 ± 7.904 and -4.649 ± 6.986, respectively (p = 0.753), while the mean PIP at baseline and after transport was 32.4 ± 5.53 and 32.73 ± 6.51, respectively (p = 0.577). The mean initial FiO_2_ was 54.68 ± 23.98, while that after transport increased significantly to 100 ± 0 (p < 0.001). While the HCO_3_, PIP, and BE values did not change significantly during transport, the mean pH, PaCO_2_, and lactate levels increased significantly to approach normal range values. Blood gas analysis of all patients is shown in Table 2.

In type 1 respiratory failure patients, the initial mean pH, PaCO_2_, HCO_3_, lactate, and BE values did not differ significantly from those after transport (pH 7.381 ± 0.146 vs. 7.366 ± 0.167, p = 0.596; PaCO2 108.43 ± 73.47 vs. 289.19 ± 154.23, p< 0.05; HCO3 21.19 ± 5.97 vs. 20.33 ± 5.64, p = 0.6; lactate 5.05 ± 4.71 vs. 4.45 ± 4.09, p = 0.306; and BE -4.55 ± 7.44 vs. -5.45 ± 7.04, p = 0.403). However, the initial PaO_2_ of type 1 respiratory failure patients (108.43 ± 73.47) increased significantly after transport (289.19 ± 154.23; p< 0.05). Blood gas analysis of type 1 respiratory failure patients is shown in Table 3.

In type 2 respiratory failure patients, the mean pH and PaO_2_ levels increased significantly (pH 7.13 ± 0.16 vs. 7.23 ± 0.15, p< 0.05; PaO_2_ 92.98 ± 78.68 vs. 259.96 ± 148.06, p< 0.05), while the mean PaCO_2_ levels decreased significantly (71.1 ± 22.18 vs. 49.85 ± 15.73, p< 0.05) between baseline and posttransport. However, in this group, the initial HCO_3_, lactate, and BE values did not differ from those after transport (HCO_3_ 18.87 ± 6.52 vs. 20.86 ± 5.69, p = 0.0681; lactate 5.64 ± 4.76 vs. 4.67 ± 4.63, p = 0.081; and BE -4.55 ± 8.48 vs. -3.81 ± 6.94, p = 0.256). Both pH and PaCO_2_ approached normal values in this group after transport. Blood gas analysis of type 2 respiratory failure patients is shown in Table 4.

Device-related complications during transport included O_2_ cable disconnection (11.6%), device failure (2.59%), vomiting (1.2%), and extubation (2.59%). Of these events, 78.5 % were due to team failure, 14.2% were due to equipment failure, and 7.1% were related to delays. No aspiration or pneumothorax was noted during transport. Device-related complications during transport are shown in Table 5.

## 4. Discussion

In-hospital transport of patients to tomography, MRI, and angiography units is a routine and essential aspect of care for critically ill, emergency service, and ICU patients. Achieving patient transport within a hospital or between medical facilities without interrupting medical treatment can be challenging [2]. Indeed, ICU ventilators are not designed for transport, and their use is limited by their size, weight, and requirement for a power supply. These limitations also restrict their use in the context of emergency medical care. Transportation of critically ill patients requiring mechanical ventilation is recognized as a high-risk and expensive procedure [3]. The equipment used for in-hospital patient transfers should be safe for the patient, inexpensive, and easy to use.

Disposable mechanical ventilators are a reasonable choice for in-hospital transfers. The LPR is a gas-powered automatic resuscitator designed for short-term ventilation during the transport of critically ill mechanically ventilated patients.

Recommendations to minimize the incidence of adverse events during patient transport include careful planning and ensuring that the following are available: a defibrillator, resuscitation equipment and drugs, sufficient oxygen supplies, a manual resuscitator with a mask, and skilled personnel, actively using LPR after 1 hour of practical training by an anesthesiologist specializing in automatic transport ventilators [4]. While the majority of adverse events during patient transport are minor, they occur in up to 68% of transfers [5]. Moreover, serious adverse events resulting in physiologic compromise requiring therapeutic intervention do occur, with a reported incidence of 4.2–8.9% [6]. In our study, a total of 14 (18.18%) complications were noted during 77 in-hospital patient transports, and the risks associated with transport were reported to be manageable by the skilled personnel.

The hemodynamic effects of mechanical ventilation are complex and may affect HR, preload, afterload, and blood pressure [7, 8]. The circulatory effects of mechanical ventilation can be dangerous during patient transport [9]. However, our study showed no significant differences in vital signs before versus after transport in critically ill mechanically ventilated patients with LPR. After transport, the mean pH, PaCO_2_, and lactate levels of all patients increased significantly and approached normal range values. The lactate levels of all patients decreased significantly and approached normal range values. Moreover, PaO_2_ values increased after transport, whereas HCO_3_ and BE values did not significantly change after transport.

Our study included both type 1 and 2 respiratory failure patients. The mean pH, PaCO_2_, HCO_3_, BE, and lactate values of type 1 respiratory failure patients did not change after transport. However, the mean PaO_2_ significantly increased. The LPR device functions with a 15 L/min continuous O_2_ flow, and increasing PaO_2_ is associated with high FiO_2_ during transport. The increase in PaO_2_ is due to the gas-powered design of the device, which allowed for safe transport of type 1 respiratory failure patients according to the blood gas analysis done in this study.

In type 2 respiratory failure patients, the mean pH increased significantly and approached normal values; while PaO_2_ values increased significantly, PaCO_2_ values decreased significantly, and HCO_3_, lactate, and BE values did not change after transport. Similar to type 1 respiratory failure patients, according to blood gas analysis, the LPR contributed to the safe transport of patients with type 2 respiratory failure.

Our study had several limitations. First, the LPR device was tested on hemodynamically stable patients and not hypotensive patients. Second, the testing duration was limited to 1 hour, and the device performance after this period was not assessed. Moreover, the device was not evaluated for use in trauma patients. Thus, the performance of the LPR in patients with lung contusions should be evaluated. Finally, no morbidly obese patients were included in the study group; further evaluation is warranted for this patient group.

In conclusion, there are various disposable mechanical resuscitator models, and institutions should choose the device that best fits their needs. In our study group, the LPR was reliable according to the vital signs and blood gas analyses, although these devices should be used only by skilled personnel due to the possible risk of complications during transport.

## Figures and Tables

**Table 1:** Vital signs of all patients (MAP: mean arterial pressure, HR: heart rate; RR: respiratory rate).

	Pre-transport	After Transport	P
	mean±SD	mean±SD	
MAP	69,16±4,78	69,09±4,18	0,898
HR	99,82±11,64	99,34±12,33	0,412
RR	14,44±2,54	14,6±2,11	0,453

**Table 2:** Blood gas analysis of all patients.

	Pre-transport		After Transport		P
	mean±SD	%95 CI of mean	mean±SD	%95 CI of mean	
pH	7,259±0,197	7,208-7,310	7,315±0,169	7,271-7,359	0,001
PaO2	100,837±75,824	81,077-120,597	274,828±150,647	235,569-314,087	4E-15
PaCO2	51,732±25,114	45,187-58,277	41,866±16,262	37,627-46,104	0,00015
HCO3	20,054±6,306	18,410-21,697	20,594±5,625	19,128-22,060	0,102
Lactate	5,340±4,705	4,114-6,566	4,566±4,331	3,437-5,694	0,047
BE	-4,554±7,904	-6,61 – (-2,494)	-4,649±6,986	-6,469-(-2,828)	0,753
FiO2	54,68±23,98	49,23-60,12	100±0	-	<0,001
PIP	32,4±5,53	31,15-33,66	32,73±6,51	31,25-34,21	0,577

**Table 3:** Blood gas analysis of type 1 respiratory failure patients.

	Initial		After Transport		P
	mean±SD	%95 CI of mean	mean±SD	%95 CI of mean	
pH	7,381±+0,146	7,327-7,436	7,366±0,167	7,303-7,428	0,596
PaO2	108,43±73,47	80,99-135,86	289,19+154,23	231,6-346,78	1E-09
PaCO2	33±6,94	30,41-35,59	34,14±12,82	29,35-38,93	0,925
HCO3	21,19±+5,97	18,95-23,42	20,33±5,64	18,22-22,44	0,6
Lactate	5,05±4,71	3,29-6,8	4,45±4,09	2,92-5,98	0,306
BE	-4,55±7,44	(-7,33)-(-1,77)	-5,45±7,04	(-8,08)-(-2,82)	0,403

**Table 4:** Blood gas analysis of type 2 respiratory failure patients.

	Initial		After Transport		P
	mean±SD	%95 CI of mean	mean±SD	%95 CI of mean	
PH	7,13±0,16	7,07-7,19	7,23±0,15	7,2-7,32	0,0000006
PaO2	92,98±78,68	63,05-122,91	259,96±148,06	203,64-316,28	0,0000002
PaCO2	71,1±22,18	62,67-79,54	49,85±15,73	43,86-55,83	0,00003
HCO3	18,87±6,52	16,39-21,36	20,86±5,69	18,7-23,03	0,0681
Lactate	5,64±4,76	3,83-7,45	4,67±4,63	2,91-6,44	0,081
BE	-4,55±8,48	-7,77)-(-1,32)	-3,81±6,94	-6,45)-(-1,17)	0,256

**Table 5:** Device-related complications during transport.

Complication	n (%)
Aspiration	0 (0%)
O2 Cable disconnection	9 (11,6%)
Displacement of the intubation tube	2 (2,59%)
Device failure	2 (2,59%)
Vomitting	1 (1,2%)
Pneumothorax	0 (0%)

## Data Availability

The SPSS/Excel data are used to store the findings of this study. Data are available from Mehmet Özgür Erdoğan, Department of Emergency Medicine, Bahcesehir University, Istanbul, Turkey, for researchers who meet the criteria for access to confidential data. Please mail us on ozgurtheerdogan@mynet.com.
